# Palliative non-small cell lung cancer treatment and end-of-life care stratified by sex and childlessness: an important interplay in unmarried patients?

**DOI:** 10.1007/s00520-022-06987-7

**Published:** 2022-03-22

**Authors:** Carsten Nieder, Siv Gyda Aanes, Ellinor C. Haukland

**Affiliations:** 1 Department of Oncology and Palliative Medicine, Nordland Hospital, 8092 Bodø, Norway; 2 Department of Clinical Medicine, Faculty of Health Sciences, University of Tromsø, Tromsø, Norway; 3 SHARE – Center for Resilience in Healthcare, Faculty of Health Sciences, Department of Quality and Health Technology, University of Stavanger, Stavanger, Norway

**Keywords:** Non-small cell lung cancer, Palliative therapy, Marital status, End-of-life care, Hospital death

## Abstract

**Purpose:**

To analyze the interplay of sex and presence of children in unmarried patients with non-small cell lung cancer, because previous studies suggested sex-related disparities. Adult children may participate in treatment decisions and provision of social support or home care.

**Methods:**

Retrospective single-institution analysis of 186 unmarried deceased patients, managed according to national guidelines outside of clinical trials. Due to the absence of other oncology care providers in the region and the availability of electronic health records, all aspects of longitudinal care were captured.

**Results:**

Eighty-eight female and 98 male patients were included, the majority of whom had children. Comparable proportions in all four strata did not receive active therapy. Involvement of the palliative care team was similar, too. Patients without children were more likely to receive systemic therapy (39% utilization in women with children, 67% in women without children, 41% in men with children, 52% in men without children; *p* = 0.05). During the last 3 months of life, female patients spent significantly more days in hospital than their male counterparts. Place of death was not significantly different. Home death was equally uncommon in each group. In the multivariate analysis, survival was associated with age and cancer stage, in contrast to sex and presence of children.

**Conclusion:**

In contrast to studies from other healthcare systems, unmarried male patients were managed in a largely similar fashion to their female counterparts and with similar survival outcome. Unexpectedly, patients without children more often received systemic anti-cancer treatment.

## Introduction

Disparities in patterns of cancer care and outcomes have received increasing attention in recent years. Demographics, socioeconomic factors, and other baseline variables might result in reduced access to treatment and/or unnecessarily poor outcomes [1]. Important factors to consider include sex and marital status. The latter was evaluated by Aizer et al. in a large Surveillance, Epidemiology and End Results (SEER; USA) program analysis [2]. Patients diagnosed in 2004 through 2008 with lung, colorectal, breast, prostate, or other common cancers were included. Married patients were significantly less likely to present with metastatic disease, more likely to receive definitive treatment and less likely to die from cancer than unmarried patients. These associations remained significant when each individual cancer was analyzed. The benefit associated with marriage was greater in males than females for all outcome measures. Also, a newer SEER analysis (2007–2016; 9 cancer types) suggested that marriage may play a greater protective role in the cancer-specific survival of men than of women [3].

Especially patients with lung cancer continue to experience poor survival, and this fact is even more pronounced in elderly and rural cohorts [4]. Previous studies in our health care region in rural Norway have described the patterns of care for non-small cell lung cancer (NSCLC) patients with focus on palliative treatment and end-of-life care [5–7]. In the curative setting of early-stage NSCLC, factors including but not limited to unmarried status, advancing age, and male sex were associated with no treatment (SEER 2004–2012) [8]. No treatment portended a worse cancer-specific survival and overall survival. These findings from previous studies prompted our group to analyze potential disparities in unmarried patients, hypothesizing that male sex also might impact on aspects of treatment that were not included in these studies, e.g., hospitalization, place of death, or referral to palliative care. Furthermore, we included the interplay of sex and presence or absence of children, because adult children as caregivers might have important roles in treatment decisions and provision of social support or home care [9].

## Patients and methods

The study included 186 unmarried, deceased patients with NSCLC diagnosed between 2006 and 2020. Focusing on deceased patients allows for evaluation of end-of-life care. On the other hand, survival analysis is imperfect, because the usual concept of censoring patients who continue follow-up cannot be applied. The patients were identified from a pre-existing quality-of-care database established by the Department of Oncology and Palliative Medicine, which captures all patients who receive lung cancer treatment in our geographical region, after exclusion of those who lived with a partner or spouse. The patients were widowed, divorced, separated, or never married. Children of all ages were permitted and coded as present or absent, regardless of number and place of living. All patients were managed in a real-life setting after the multidisciplinary lung tumor board at Nordland Hospital Bodø had provided the treatment strategy, which always was based on national Norwegian guidelines. All initial stages of NSCLC and management approaches were included (best supportive care, systemic treatment, radiotherapy, surgery). Patients managed with curative intent who relapsed and eventually died from recurrent disease were included, while those without relapse who were still alive were excluded. Due to the absence of other oncology care providers in the healthcare region and the availability of electronic health records, all aspects of longitudinal NSCLC care were captured, and the study resembled a cancer registry study, albeit with limited number of patients. Previous studies with identical preconditions have already elaborated on the completeness of sociodemographic and management data and other advantages of the quality-of-care surveillance in our geographical region [5–7]. All patients were covered by the national public healthcare system. Therefore, no financial barriers prevented access to hospital or nursing home care, radiotherapy, drugs etc.

### Statistical analysis and ethics

Baseline and treatment data were extracted and compared between different subgroups. The IBM SPSS 27 software package (IBM SPSS Statistics, Somers, NY, USA) was employed for all statistical analyses. For comparison of dichotomous variables, the chi-square test and Fisher’s exact test, where applicable, were employed, and for continuous variables, the Mann–Whitney *U* test. Significance level was set to 5%, and all tests were carried out two-sided. The Kaplan–Meier method was used to analyze overall survival and the log-rank test to compare survival curves. A forward conditional Cox regression analysis was performed to analyze the impact of sex and presence of children, while considering age, histology, and cancer stage. As noted earlier, the survival outcomes slightly underestimate the true survival of all patients in our database. Due to the high incidence of stage III and IV disease in our region and the long time period of inclusion (2006–2020), only a limited number of living patients would have been censored in a complete analysis. The study was performed as a retrospective analysis in the context of our already approved longitudinal monitoring of NSCLC management. Additional approval from the Regional Committee for Medical and Health Research Ethics (REK) was not necessary for this project, which already had exempt status.

## Results

### Study population

The study included 88 unmarried female (47%) and 98 unmarried male patients (53%). Among female patients, 76 (86%) had children and 12 (14%) had none. Also, the majority of men had children (*n* = 63, 64%), as displayed in Table 1. The majority of unmarried patients without children had male sex (35 of 47, 74%). The female patients were slightly older (median 73 (range 45–90) versus 69 years (42–89), *p* = 0.10. Age did not significantly impact on the presence of children, regardless of sex. Distance to hospital was not significantly correlated with sex and presence of children. The same was true for the number of prescription drugs used immediately before lung cancer diagnosis (median 2.5–3.0), a surrogate for comorbidity. Active smoking at lung cancer diagnosis was more common in men (63% if no children, 49% if children) than women (33 and 35%, respectively), *p* = 0.06. Men were more often diagnosed with squamous cell cancer (42% compared to 27% in women, *p* = 0.05). Stage was not equally distributed either (IV in 40% of men without children, but 60–67% in all other groups; IIIB and IV in 54% of men without children, but 73–75% in all other groups; *p* = 0.06).

**Table 1:** Baseline characteristics for 186 deceased unmarried patients with non-small cell lung cancer included in the study

Parameter	Female, no children	Female, children	Male, no children	Male, children
Group size	12	76	35	63
Median age, years	74	73	68	69
Squamous cell cancer	4 (33%)	20 (26%)	15 (43%)	26 (41%)
Non-squamous cell cancer	8 (67%)	56 (74%)	20 (57%)	37 (59%)
Primary stage IV	8 (67%)	47 (62%)	14 (40%)	38 (60%)
Primary stage III or less	4 (33%)	29 (38%)	21 (60%)	25 (40%)
Brain metastases*	3 (25%)	20 (26%)	8 (23%)	17 (27%)
Diabetes mellitus	1 (8%)	7 (9%)	7 (20%)	6 (10%)
Previous cancer history**	4 (33%)	9 (12%)	5 (14%)	12 (19%)
Active smoker	4 (33%)	27 (35%)	22 (63%)	31 (49%)

^*^Combined (at primary cancer diagnosis or later during the disease trajectory)

^**^Other than non-small cell lung cancer (often bowel, kidney or skin cancer)

### Treatment

No active treatment was pursued in 17% of women with children (16% of men with children) and 17% of women without children (9% of men with children), *p* > 0.2. No care by the multidisciplinary palliative care team was received by 48% of women with children (47% of men with children) compared to 30% of women without children (45% of men without children). Regarding the two subgroups of female patients, the *p*-value was > 0.2. Female and male patients were equally likely to receive systemic anti-cancer treatment. Patients without children were more likely to receive systemic therapy (39% utilization in women with children, 67% in women without children, 41% in men with children, 52% in men without children; *p* = 0.05). The rates of active treatment during the last 4 weeks of life were comparable (26–33%, *p* > 0.2).

During the last 3 months of life, female patients spent significantly more days in hospital than their male counterparts, median 20 (range 0–53) versus 14 (range 0–40), *p* = 0.004. Identical results were obtained in women with/without children (20 days each), and comparable results in men (14.5 days with and 12.5 days without children), *p* > 0.2. Place of death was not significantly different either. Home death was equally uncommon in each group (≤ 11%). Somewhat larger numerical differences were observed for hospital death (36% in women with children, 39% in men with children, 27% in women without children, and 25% in men without children, respectively). Many patients died in primary health care institutions such as nursing homes. The number of days spent in these institutions during the last 3 months of life was highest in men without children (median 23 compared to 7–11 in the other groups). However, none of these differences were statistically significant or close to it. Data regarding utilization of home care or other community/primary healthcare services were not available.

### Survival

**Fig. 1:**
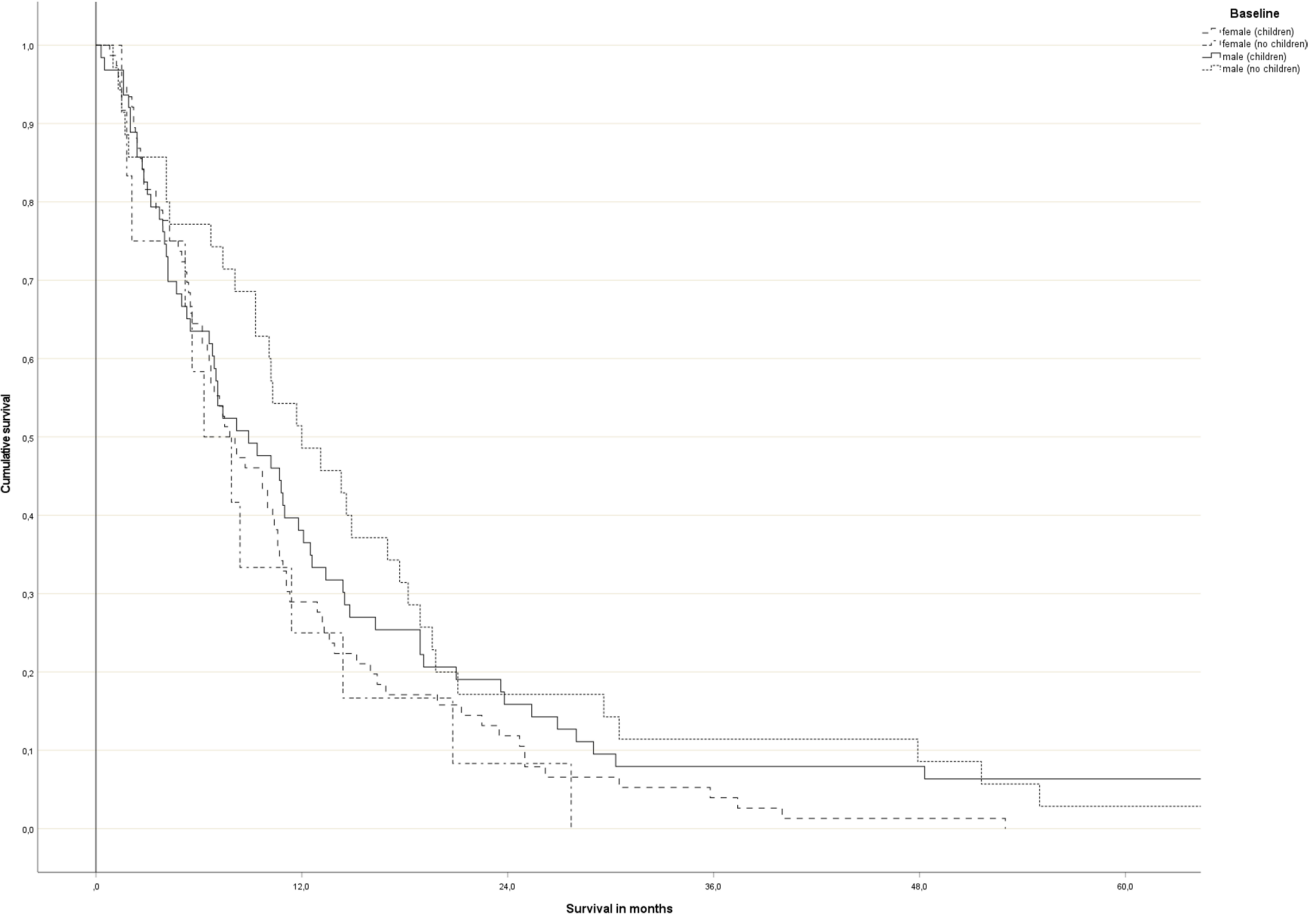
Actuarial Kaplan–Meier curves stratified for sex and presence of children. Level of significance (log-rank test): *p* = 0.036 (male patients without children versus female patients with children), *p* = 0.088 (male patients without children versus female patients without children), *p* > 0.1 (other pairs)

Median overall survival was 9.3 months (female patients: 7.8 months, male patients: 10.2 months, *p* = 0.03). The difference between patients with and without children was not significant (median 8.2 versus 10.3 months, *p* > 0.2). Figure 1 shows the survival curves for all 4 strata. Male patients without children had the longest median survival (12.0 months).

Eligibility for different initial treatment strategies, which reflects factors such as stage and comorbidity to name a few, resulted in clearly distinct outcomes. For example, median survival after best supportive care was 4 months (systemic platinum-doublet chemotherapy 8 months, palliative radiotherapy with total dose ≤ 30 Gy 7 months, palliative radiotherapy with higher total dose 16 months, curative radio(chemo)therapy 25 months).

In the multivariate analysis, age as continuous variable (*p* = 0.002) and stage as dichotomized variable (IIIB and IV versus others, *p* < 0.0001) were significant, in contrast to histology, sex and presence of children, as displayed in Table 2.

**Table 2:** Multivariate forward conditional Cox regression analysis, endpoint: overall survival

Parameter	Hazard ratio	95% confidence interval	*p*-value
Age, continuous	1.02	1.01–1.03	0.002
Stage, ref. I-IIIA (versus IIIB/IV)	0.62	0.49–0.77	< 0.0001
Histology, ref. squamous cell (versus non-squamous)	1.30	0.94–1.64	0.26
Sex, ref. female (versus male)	1.09	0.88–1.29	0.30
Children, ref. present (versus none)	0.79	0.41–1.18	0.40

## Discussion

In a previous, large-scale SEER study (2004–2009) of patients with NSCLC, being unmarried was associated with significantly decreased cancer-specific survival (hazard ratio (HR): 1.14, 95% confidence interval (CI): 1.12–1.17, *p* < 0.001) [10]. Among the unmarried group, patients who were single had worse cancer-specific survival (median survival 12 months) than those who were divorced (median survival 15 months, *p* < 0.001) or widowed (median survival 15 months, *p* < 0.001). It is not known from this and comparable previous studies [2, 8] whether or not other family members or relatives acted as caregivers. Hypothetically, having children might be advantageous for unmarried cancer patients, unless social and practical support from other sources compensates for family-derived assistance [11]. In principle, a 65-year-old, recently widowed grandmother with 2 children and 3 grandchildren may have a social network tremendously different from that of a never-married, childless, single man. In other words, large heterogeneity may be present in populations of unmarried patients with NSCLC, yet limited knowledge exists about the impact of being childless. Therefore, the present study stratified unmarried patients (defined as neither living with a partner nor spouse) by presence/absence of children and sex, another factor that has been correlated with outcome [2, 8].

According to the Norwegian cancer registry, lung cancer is more common in males [12], a finding also reflected in the present study. Regarding the study population (largely patients in their sixties and older), relevant findings included that the majority of unmarried patients without children had male sex (35 of 47, 74%). Furthermore, active smoking at lung cancer diagnosis was more common in men (63% if no children, 49% if children) than women (33 and 35%, respectively). Men were significantly more often diagnosed with squamous cell cancer (42% compared to 27% in women). Stage was not equally distributed either (IV in 40% of men without children, but 60–67% in all other groups; IIIB and IV in 54% of men without children, but 73–75% in all other groups).

With regard to aspects of treatment, few if any statistically significant disparities were identified. Surprisingly, patients without children were more likely to receive systemic therapy. The presence of an otherwise well-functioning social network coupled with access to community-based oncology nurses, if needed and agreed to by the patients, may explain that these patients did not receive less treatment, as one might have anticipated from data derived from healthcare systems with greater barriers to supportive care. Across all 4 strata, active treatment during the last 4 weeks of life was relatively common (26–33%). Less than 50% of the patients had contact with the hospital-based palliative care team at some time during treatment, probably a sub-optimal utilization. Female patients spent significantly more days in hospital than their male counterparts, median 20 versus 14 days during the last 3 months of life, and this indicator was not modified by childlessness. Across all 4 strata, a majority of patients died in nursing homes and few (≤ 11%) at home. In a SEER-Medicare database study, decedents diagnosed with lung cancer at age ≥ 66 years between January 2007 and December 2013 who survived ≥ 6 months from diagnosis were included [13]. Between 6 and 1 month before death, full-month hospice and inpatient/skilled nursing increased. Cancer-directed treatment decreased from 31.9 to 18.5%. The percentage receiving such treatment was higher for males, unmarried, and younger age. Associations between sociodemographic characteristics and care setting suggest differences in care preferences or access barriers, in line with other SEER studies [8, 10]. In a UK study, no association was found between aggressive end-of-life care (greater than or equal to one of the following indicators occurring during the last 3 months of life: greater than or equal to two emergency department visits, ≥ 30 days in hospital and death in hospital) and patients’ age, gender, marital, and financial or health status [14]. Overall, results from different countries with different healthcare systems were heterogeneous.

As evident from our multivariate analysis of overall survival, the repeatedly reported result of inferior survival in male patients could not be confirmed in the present study. Furthermore, there was no relevant impact of childlessness. Comparison to the literature is hampered by differences in study design and patient selection, including the fact that cancer survivors did not enter our study (actual survival is therefore better than reported). The issue of different methods also applies to a Japanese study showing that childless patients had significantly shorter survival in comparison with patients with children [15]. However, these were NSCLC patients treated with surgery who were not selected by marital status. Radkiewicz et al. performed a nationwide, population-based cohort study in Sweden using data on all incident cases of lung squamous cell carcinoma (*n* = 10,325) and adenocarcinoma (*n* = 23,465), i.e., NSCLC, recorded in 2002–2016 [16]. They computed adjusted female-to-male hazard ratios (aHRs) and standardized survival proportions over follow-up including, e.g., age, marital status, and comorbidities, but not childlessness. Women presented with better performance status, were younger, and more often never-smokers. Men with adenocarcinoma had a consistently poorer lung cancer–specific survival across stage; HR 0.94; 95% CI 0.88–0.99 (stage IIIB-IV), remaining largely unchanged after adjustments. The same pattern was observed in squamous cell carcinoma, except in stage IIIA disease, where the Swedish study found no sex differences in survival.

The limitations of the present study must be considered, when trying to perform comparisons, especially with the much larger nationwide studies. Due to higher statistical power, these large studies might reveal significant correlations where our study detected numerical differences with *p* > 0.05. As mentioned previously, disparities may become less likely if a national healthcare system with universal coverage and easy access to care aims at providing equal access, such as in Norway or Sweden. Massive differences exist to the US healthcare system, where income and insurance status impact on access to care, and high out-of-pocket payments create barriers for a considerable proportion of the population.

Despite its numerous advantages, our database is not perfect and lacks, e.g., longitudinal symptom burden, performance status, and background of marital status (widowed, divorced, never married etc.). We did not account for number of children, their place of living, other relatives, or the complete social network. In Norway, the primary health care sector and social welfare system are able to provide considerable support to singles with cancer and other serious diagnoses. The fact that we included patients managed between 2006 and 2020 means that available treatment options have evolved. For example, immune checkpoint inhibitors were not available in the earlier years of the study. In contrast, in- and out-patient support, e.g., access to palliative care, palliative radiotherapy, nursing home admission, and home care, has remained stable. Overall, our data did not reveal major concerns regarding any subgroup, but it appears relevant to deepen the aspect of hospitalizations or total length of stays during the last 3 months of life, including reason for admission and possible waiting time for available nursing home care at preferable date of discharge (unplanned prolongation of hospital stay). Thus, additional and preferably larger studies are warranted.

## Data Availability

Data can be requested for the purpose of pooled or meta-analyses by contacting the corresponding author.
